# Annotations

**Published:** 1892-09-24

**Authors:** 


### Annotations.

**Damp Towels.**

On the whole, a man who puts a damp towel round his head to keep him awake and at work after midnight is a fool. Every medical student has two objects before him: first, his immediate object of college distinction; and secondly, his after object of worthy success in life. One of these is greater than the other! Whether is the greater? Surely, it is greater to win a worthy success in life than to win high distinction at college. "But," says the student, "I must win distinction at college in order to win high success in life." That is true, so far as it goes, but it does not go very far. The way to look at the question is to consider the whole of life as one race, and that a long one. Of this long race the few years at college form a brief part. Now, every man who intends to win a long race begins steadily and soberly. If he were to put on all his steam at first, he would lose to a dead certainty. Is a man, then, not to aim at college distinctions? Of course he is! But if he be a man of real intellectual capacity, he will aim at such distinctions as he can win without damaging his brain and his general health. Every man discovers sooner or later if he lives long enough, that a good stomach, sound sleeping powers, freshness of brain, and plenty of physical capacity for work, are factors that are absolutely indispensable to success in actual life. Without all these, or most of them, college honours are as flat and useless as damp gunpowder on the battle-field. There is a secret which once discovered usually leads to both college distinction and the preservation of fresh and cheerful health. The secret is to work with might and main when one is at it; and then to play and enjoy life with equal zest when a reasonable amount of good work has been done and completed every day. This is an old, old secret, truly. But then bread is an old institution, and so is water; but nobody ever seriously proposes to invent anything newer and better.

**Aristotle's Method of Study.**

The General Medical Council of Great Britain has in its wisdom resolved that no man shall in future gain a qualification for the practice of medicine without first devoting himself to study and discipline for five years. Within the memory of our grandfathers, any man who so chose could practice physic without any study at all. The contrast between then and now is striking, and invites consideration. The Council, it is evident, is resolved that medical students shall not only receive a medical education, but shall in deed and in truth be educated men. Many students no doubt think this insistence upon an extra year of study, and the consequent "stiffening" of examinations, a serious hardship. As a matter of fact, the Council could not have done a kinder or a more paternal thing. It has thrown out a challenge to medical students. It says to them, in effect—Here is a grand feast of real knowledge placed before you; of knowledge of things, deep things, things scientific, things practical, things interesting, things which have engaged the minds of the greatest thinkers ever since men began to think. It is not a feast of words, of mere grammar, or rhetoric, or logic; but of actual things which have been from the beginning, and which will always be! Fall to—understand them, master them, make them your own for ever. That is the invitation of the General Medical Council. It is certain that the majority of medical students of the present day will respond to this invitation with alacrity. They are extremely willing to sit down to the feast of knowledge, and extremely anxious to enjoy it, and grow mentally rich, strong, and capable thereby. Is there any method which will be generally serviceable to all students of medicine and allied science? There is; it is the method of Aristotle! The Stagirite was, on the whole, the most profoundly learned of all men who have ever devoted themselves to material science. He had one method, and one only. Here it is, as condensed and summarised by Sir Alexander Grant, one of the latest, as he was certainly one of the most accomplished of Aristotle's many editors. "This method," says Sir Alexander, "consists in concentration of the mind upon the subject in hand, marshalling together all the facts and opinions attainable upon it, and dwelling on these and scrutinising and comparing them till a light flashes on the whole subject. Such is the procedure to be learnt, by imitation, from Aristotle." An exceedingly simple procedure, says the inexperienced student! Most true! But by means of it Aristotle became—Aristotle! And every man who uses it aright can by no means fail of a very real and worthy success in life. Give it an earnest trial.

**Stands Scotland Where it Did?**

A former student of Glasgow University writes to the *Glasgow Herald* pointing out a fact which the authorities of Scottish medical schools have been trying to shut their eyes to, but which cannot longer be ignored, viz., that in Scotland the number of medical students is decreasing, while the attendance at the London schools is increasing year by year. The causes of this decline are, in his opinion, twofold—an increasing energy in the London hospitals and their teachers, and a corresponding apathy in hospital authorities in Scotland. The writer speaks especially of the Western Medical School, where he formerly studied; but others besides this have been too willing to rest on their oars, and trust to their old repute and their good systematic teaching to bring students to their doors. But every year tends more and more to develop the importance of practical clinical teaching in the hospital, and while this requires to be built up on a good foundation of sound systematic knowledge, it cannot be too ample and well organised. With this end in view, the *Glasgow Herald* correspondent makes several suggestions which are worth noting, and not by the authorities of the Western Infirmary alone. One of these is that the wards of the infirmary should be thrown open to students for a longer period than is now done. The time allowed in the wards of the Western is peculiarly limited, and this suggestion, which touches on an old grievance, is more than any of the others a special and particular one. But the following is of general value: "Let the dispensary (the out-patient department) be reserved for the senior students. They are in a position to learn there from the necessarily brief remarks of the teachers that rapid decision in diagnosis and treatment which is essential to the success of their future work in actual practice, while the juniors, who make little of it and by their presence crowd out the seniors,, would be much better confined to the wards, where they learn dressing, &c., and listen to the full and detailed description of cases possible there." A fairly long course of experience in the out-patient room would save many blunders in the early days of practice, for the out-patients give a far better idea of diseases as they are generally met with in ordinary practice, than the selected and typical cases admitted into the wards. And moreover, the greater freedom enjoyed by the out-patients will frequently give examples of that power of evading and misinterpreting doctor's orders which so many people possess, and which, with the resulting apparent failure of the means of cure so often bewilder the young practitioner. In-patients may demonstrate disease, but out-patients show human nature; and a knowledge of this is as essential to a success as even technical skill. We hope that the Western Infirmary will awake to an appreciation of this and the other suggestions of its former students; not, indeed, that it may lessen the numbers of London students, but that those who still go to it may go forth into the world as fully equipped as their brethren of the south.

